# Children with extended oligoarticular and polyarticular juvenile idiopathic arthritis have alterations in B and T follicular cell subsets in peripheral blood and a cytokine profile sustaining B cell activation

**DOI:** 10.1136/rmdopen-2022-002901

**Published:** 2023-08-31

**Authors:** Catarina Tomé, Filipa Oliveira-Ramos, Raquel Campanilho-Marques, Ana F. Mourão, Sandra Sousa, Cláudia Marques, Ana T. Melo, Rui L. Teixeira, Ana P. Martins, Sofia Moeda, Patrícia Costa-Reis, Rita P. Torres, Matilde Bandeira, Helena Fonseca, Miroslava Gonçalves, Maria J. Santos, Luis Graca, João E. Fonseca, Rita A. Moura

**Affiliations:** 1 Instituto de Medicina Molecular João Lobo Antunes, Faculdade de Medicina, Universidade de Lisboa, Centro Académico de Medicina de Lisboa, Lisbon, Portugal; 2 Rheumatology Department, Hospital de Santa Maria, Centro Hospitalar Universitário Lisboa Norte, EPE, Centro Académico de Medicina de Lisboa, Lisbon, Portugal; 3 Rheumatology Department, Hospital de São Francisco Xavier, Centro Hospitalar Lisboa Ocidental, EPE, Lisbon, Portugal; 4 Reumatology Department, Hospital Garcia de Orta, EPE, Almada, Portugal; 5 Pediatric Surgery Department, Hospital de Santa Maria, Centro Hospitalar Universitário Lisboa Norte, EPE, Lisbon, Portugal; 6 Department of Pediatrics, Hospital de Santa Maria, Centro Hospitalar Universitário Lisboa Norte, EPE, Centro Académico de Medicina de Lisboa, Lisbon, Portugal

**Keywords:** Juvenile Idiopathic Arthritis, B cells, Cytokines, T-Lymphocyte subsets

## Abstract

**Objectives:**

The main goal of this study was to characterise the frequency and phenotype of B, T follicular helper (Tfh) and T follicular regulatory (Tfr) cells in peripheral blood and the cytokine environment present in circulation in children with extended oligoarticular juvenile idiopathic arthritis (extended oligo JIA) and polyarticular JIA (poly JIA) when compared with healthy controls, children with persistent oligoarticular JIA (persistent oligo JIA) and adult JIA patients.

**Methods:**

Blood samples were collected from 105 JIA patients (children and adults) and 50 age-matched healthy individuals. The frequency and phenotype of B, Tfh and Tfr cells were evaluated by flow cytometry. Serum levels of APRIL, BAFF, IL-1β, IL-2, IL-4, IL-6, IL-10, IL-17A, IL-21, IL-22, IFN-γ, PD-1, PD-L1, sCD40L, CXCL13 and TNF were measured by multiplex bead-based immunoassay and/or ELISA in all groups included.

**Results:**

The frequency of B, Tfh and Tfr cells was similar between JIA patients and controls. Children with extended oligo JIA and poly JIA, but not persistent oligo JIA, had significantly lower frequencies of plasmablasts, regulatory T cells and higher levels of Th17-like Tfh cells in circulation when compared with controls. Furthermore, APRIL, BAFF, IL-6 and IL-17A serum levels were significantly higher in paediatric extended oligo JIA and poly JIA patients when compared with controls. These immunological alterations were not found in adult JIA patients in comparison to controls.

**Conclusions:**

Our results suggest a potential role and/or activation profile of B and Th17-like Tfh cells in the pathogenesis of extended oligo JIA and poly JIA, but not persistent oligo JIA.

**WHAT IS ALREADY KNOWN ON THIS TOPIC**

- Disturbances in adaptive immune responses have been implicated in the development of oligoarticular and polyarticular juvenile idiopathic arthritis (JIA). Evidence from the literature suggests that B cells might have a relevant role in the pathogenesis of JIA. Nevertheless, the knowledge about follicular T cells in JIA development is still scarce.

**WHAT THIS STUDY ADDS**

- In this study, we have extensively characterised B, T and follicular T cell subsets in peripheral blood, as well as evaluated a wide panel of proinflammatory and anti-inflammatory cytokines in paediatric and adult patients with extended oligoarticular and polyarticular JIA, an issue not addressed before in detail. Our study suggests that alterations in circulating B cells and follicular T cell subsets, particularly Th17-like T follicular helper cells, might be associated to the pathogenesis of extended oligoarticular JIA and polyarticular JIA, in line with previous observations obtained in adults with early rheumatoid arthritis (RA), thus reinforcing clinical evidence showing that these JIA categories tend to evolve into a RA like phenotype.

**HOW THIS STUDY MIGHT AFFECT RESEARCH, PRACTICE OR POLICY**

- Our findings provide insights about the immunopathogenesis of extended oligoarticular JIA and polyarticular JIA in terms of B and T cell immunity when compared with persistent oligoarticular JIA, healthy individuals and adult JIA patients. These results pave the way for further studies on B and follicular T cell immune responses in JIA, which might contribute to the optimisation of JIA treatment options.

## Introduction

Juvenile idiopathic arthritis (JIA) is the most common rheumatic disorder in children.^1^ The term JIA embraces a clinically heterogeneous group of chronic immune-mediated arthritides of unknown aetiology that, by definition, affects children younger than 16 years of age and lasts for at least 6 weeks.^2 3^ JIA can lead to both short-term and long-term morbidity and physical disability, which significantly compromises the quality of life of patients and negatively impacts their life expectancy.^4–6^ Despite the recent progress achieved in JIA treatment, active disease persists into adulthood in a substantial percentage of patients, which underlines the importance to investigate JIA heterogeneity and the mechanisms associated to disease immunopathogenesis.^7 8^ The International League of Associations for Rheumatology (ILAR) criteria define seven categories of JIA: oligoarticular (persistent or extended), polyarticular rheumatoid factor (RF)-negative (RF−), polyarticular RF positive (RF+), systemic, enthesitis related arthritis, psoriatic arthritis and undifferentiated arthritis.^9^ Recently, our group has demonstrated that extended oligoarticular JIA (extended oligo JIA) and polyarticular JIA (poly JIA) patients mostly evolve to a rheumatoid arthritis (RA) like phenotype in adulthood with an equally poor outcome.^5^ In addition, we have previously shown that disturbances in B cell immune responses are implicated in RA pathogenesis since the first weeks of disease development.^10–14^ Evidence from the literature support that both innate and adaptive immune responses are implicated in JIA development.^15–17^ B cells have important roles in JIA pathogenesis through autoantibody production, antigen presentation, cytokine release and/ or T cell activation.^18^ The study of B cells has not been extensively explored in JIA, but it has been suggested that B cells might have a relevant role in JIA pathophysiology.^18–20^ In fact, altered B cell homeostasis, B cell differentiation and B cell hyperactivity have been described in JIA.^21–27^ The detection of autoantibodies such as antinuclear antibodies (ANA), RF and anti-citrullinated protein antibodies (ACPA) in JIA patients supports a breakdown in B cell tolerance.^28^ Importantly, the effectiveness of B cell depletion therapy with rituximab in JIA further supports B cell intervention in the disease pathophysiology.^29–31^ Follicular T helper cells (Tfh) and follicular T regulatory cells (Tfr) have been described as critical players for the initiation, maintenance and regulation of germinal centre (GC) reactions.^32 33^ Tfh cells help B cells by supporting antibody affinity maturation, class-switch recombination and differentiation of long-lived plasma cells and memory B cells, whereas Tfr cells regulate Tfh cell-mediated immune responses.^34–36^ Recent studies by our group and others have demonstrated that a dysregulated function of Tfh and Tfr cells and/or their inadequate balance are associated to the pathogenesis of several autoimmune diseases of typically adult onset,^37–39^ including RA.^40 41^ However, the impact of Tfh and Tfr cells immune dysfunction in the pathogenesis of juvenile autoimmune disorders, such as JIA, is largely unknown. Therefore, we hypothesise that, as observed in RA, abnormal B cell immune responses are found in children with extended oligo JIA and poly JIA, and that Tfh and/or Tfr cells play a role in the alterations observed in B cell immune responses in JIA. Thus, the main goal of this study was to characterise the frequency and phenotype of B, Tfh and Tfr cells in peripheral blood and the cytokine environment present in circulation in children with extended oligo JIA and poly JIA when compared with healthy controls, children with persistent oligoarticular JIA (persistent oligo JIA) and adult JIA patients.

## Results

### Clinical characterisation of patients

A total of 105 JIA patients (children and adults) fulfilling the ILAR criteria^9^ and 50 age-matched healthy individuals were included in the present study. JIA patients were subdivided into two main groups: a group of paediatric JIA patients (n=68, 79% female), which had a mean age of 11±4 years old, a mean disease duration of 6±4 years and a mean juvenile arthritis disease activity score 27-joint reduced count (JADAS-27) score of 2.8±4.1; and a group of adult JIA patients (n=37, 84% female), which had a mean age of 27±12 years old, a mean disease duration of 18±12 years and a disease activity score of 28 joints (DAS28) of 3.3±1.5. The paediatric JIA patients were further subdivided into two study groups: persistent oligoarticular JIA (persistent oligo JIA) (n=34, 76% female) and extended oligoarticular plus polyarticular JIA (extended oligo+poly JIA) (n=34, 82% female). All patients included in this study had been treated with non-steroidal anti-inflammatory drugs (NSAIDs), synthetic and/or biologic disease-modifying anti-rheumatic drugs (DMARDs). Demographic and clinical data from all patients and healthy volunteers included in this study are described in table 1.

**Table 1:** Clinical characterisation of patients with JIA

	HC (n=27)	Paediatric JIA			P value	HC (n=23)	Adult JIA			P value
		Total JIA (n=68)	poJIA (n=34)	eo+pJIA (n=34)			Total JIA (n=37)	poJIA (n=11)	eo+pJIA (n=26)	
Demographic characteristics										
Age (years)	11±4	11±4	11±4	11±4	0.9540*	24±3	27±12	21±4	31±13	0.0056§
Sex (% female)	59% (16/27)	79% (54/68)	76% (26/34)	82% (28/34)	0.1135§	74% (17/23)	84% (31/37)	81% (9/11)	85% (22/26)	0.6372
Clinical features										
Disease duration (years)	NA	6±4	5±4	6±4	0.2365‡	NA	18±12	14±7	21±14	0.2643‡
Swollen joints (1–28)	NA	1±2	0±1	1±2	0.3397‡	NA	2±4	1±1	3±5	0.1917‡
Tender joints (1–28)	NA	1±2	0±1	1±3	0.5812‡	NA	2±4	1±1	3±4	0.1531‡
Patient VAS (1–10)	NA	1.0±1.6	0.7±1.3	1.2±1.8	0.0793‡	NA	2.0±2.3	1.2±1.3	2.3±2.6	0.5281‡
Disease activity (JADAS-27)	NA	2.8±4.1	2.1±3.0	3.5±4.8	0.3162‡	NA	NA	NA	NA	NA
Disease activity (DAS28)	NA	NA	NA	NA	NA	NA	3.3±1.5	2.9±0.8	3.5±1.6	0.5582‡
Active disease (%)	NA	47% (27/58)	41% (11/27)	52% (16/31)	0.4077§	NA	57% (17/30)	50% (4/8)	59% (13/22)	0.6568§
Low disease activity (%)	NA	9% (5/58)	4% (1/27)	13% (4/31)	0.6266§	NA	3% (1/30)	13% (1/8)	0% (0/22)	NA
Moderate disease activity (%)	NA	19% (11/58)	19% (5/27)	19% (6/31)	0.6266§	NA	40% (12/30)	37% (3/8)	41% (9/22)	NA
High disease activity (%)	NA	19% (11/58)	19% (5/27)	19% (6/31)	0.6266§	NA	13% (4/30)	0% (0/8)	18% (4/22)	NA
Remission (%)	NA	53% (31/58)	59% (16/27)	48% (15/31)	0.6266§	NA	43% (13/30)	50% (4/8)	41% (9/22)	0.6568§
Laboratory parameters										
ACPA(+)%	ND	2% (1/62)	0% (0/30)	3% (1/32)	0.3290§	ND	14% (5/35)	0% (0/11)	19% (5/26)	0.1559§
ANA(+)%	ND	54% (37/68)	65% (22/34)	44% (15/34)	0.0883§	ND	30% (11/37)	45% (5/11)	23% (6/26)	0.3314§
RF(+)%	ND	1% (1/68)	0% (0/34)	3% (1/34)	0.3602§	ND	33% (12/36)	0% (0/11)	46% (12/26)	0.0320§
CRP (mg/dL)	ND	0.21±0.40	0.18±0.30	0.25±0.48	0.3652‡	ND	0.71±1.04	0.36±0.43	0.86±1.17	0.4226‡
ESR (mm/1^st^ hour)	ND	16.9±14.7	18.6±14.6	15.3±14.8	0.207‡	ND	25.6±17.3	21.80±9.32	27.1±19.6	0.5831‡
Treatment										
Without treatment (%)	100% (0/27)	0% (0/68)	0% (0/34)	0% (0/34)	–	100% (0/23)	0% (0/37)	0% (0/11)	0% (0/26)	–
Off treatment (%)	NA	24% (16/68)	32% (11/34)	18% (6/34)	0.0931§	NA	5% (2/37)	9% (1/11)	4% (1/26)	0.6019§
NSAIDs (%)	NA	21% (14/68)	24% (8/34)	18% (6/34)	–	NA	19% (7/37)	27% (3/11)	15% (4/26)	–
Corticosteroids (%)	NA	7% (5/68)	3% (1/34)	12% (4/34)	0.0931§	NA	35% (13/37)	18% (2/11)	42% (11/26)	0.6019§
Synthetic DMARDs (%)	NA	66% (45/68)	56% (19/34)	74% (25/34)	0.0931§	NA	78% (29/37)	64% (7/11)	85% (22/26)	0.6019§
Methotrexate (%)	NA	65% (44/68)	56% (19/34)	70% (24/34)	–	NA	62% (23/37)	54% (6/11)	65% (17/26)	–
Leflunomide (%)	NA	3% (2/68)	0% (0/34)	6% (2/34)	–	NA	0% (0/37)	0% (0/11)	0% (0/26)	–
Sulfasalazine (%)	NA	0% (0/68)	0% (0/34)	0% (0/34)	–	NA	11% (4/37)	0% (0/11)	15% (4/26)	–
Hydroxychloroquine (%)	NA	0% (0/68)	0% (0/34)	0% (0/34)	–	NA	3% (1/37)	0% (0/11)	4% (1/26)	–
Cyclosporine (%)	NA	0% (0/68)	0% (0/34)	0% (0/34)	–	NA	3% (1/37)	9% (1/11)	0% (0/26)	–
Biologic DMARDs (%)	NA	25% (17/68)	18% (6/34)	32% (11/34)	0.0931§	NA	24% (9/37)	18% (2/11)	27% (7/26)	0.6019§
Etanercept (%)	NA	15% (10/68)	9% (3/34)	21% (7/34)	–	NA	8% (3/37)	0% (0/11)	12% (3/26)	–
Adalimumab (%)	NA	10% (7/68)	9% (3/34)	12% (4/34)	–	NA	8% (3/37)	9% (1/11)	8% (2/26)	–
Infliximab (%)	NA	0% (0/68)	0% (0/34)	0% (0/34)	–	NA	3% (1/37)	9% (1/11)	0% (0/26)	–
Tocilizumab (%)	NA	0% (0/68)	0% (0/34)	0% (0/34)	–	NA	3% (1/37)	0% (0/11)	4% (1/26)	–
Abatacept (%)	NA	0% (0/68)	0% (0/34)	0% (0/34)	–	NA	3% (1/37)	0% (0/11)	4% (1/26)	–
Baricitinib (%)	NA	0% (0/68)	0% (0/34)	0% (0/34)	–	NA	3% (1/37)	0% (0/11)	0% (1/26)	–

Values are represented as mean ± standard deviation. Cut-off values of JADAS-27 ≤1 and DAS28 ≤2.6 were used to define inactive disease. All patients seropositive for ACPA and/ or RF were classified as polyarticular JIA. Cut-off values used for autoantibody detection were: ANA ≥ 1/80; ACPA ≥ 20 IU/mL; RF ≥ 14 IU/mL.

Differences were considered statistically significant for p<0.05.

*Kruskal-Wallis test with post hoc Dunn’s multiple comparisons for comparisons between three independent groups (HC vs poJIA vs eo+pJIA).

§Categorical variables were analysed using χ2 test (poJIA vs eo+pJIA).

‡Continuous variables were analysed using non-parametric Mann-Whitney U test for comparisons between two independent groups (poJIA vs eo+pJIA).

ACPA, anti-citrullinated protein antibodies; ANA, antinuclear antibodies; CRP, C-reactive protein; DAS28, disease activity score of 28 joints; DMARDs, disease-modifying anti-rheumatic drugs; eo+pJIA, extended oligoarticular and polyarticular JIA; ESR, erythrocyte sedimentation rate; HC, healthy controls; JADAS-27, juvenile arthritis disease activity score 27-joint reduced count; JIA, juvenile idiopathic arthritis; NA, not applicable; ND, not determined; NSAIDs, non-steroidal anti-inflammatory drugs; PDN, prednisone; poJIA, persistent oligoarticular JIA; RF, rheumatoid factor; VAS, visual analogue scale.

### Children with extended oligoarticular and polyarticular JIA, but not persistent oligoarticular JIA patients, have lower levels of plasmablasts in circulation

**Figure 1:**
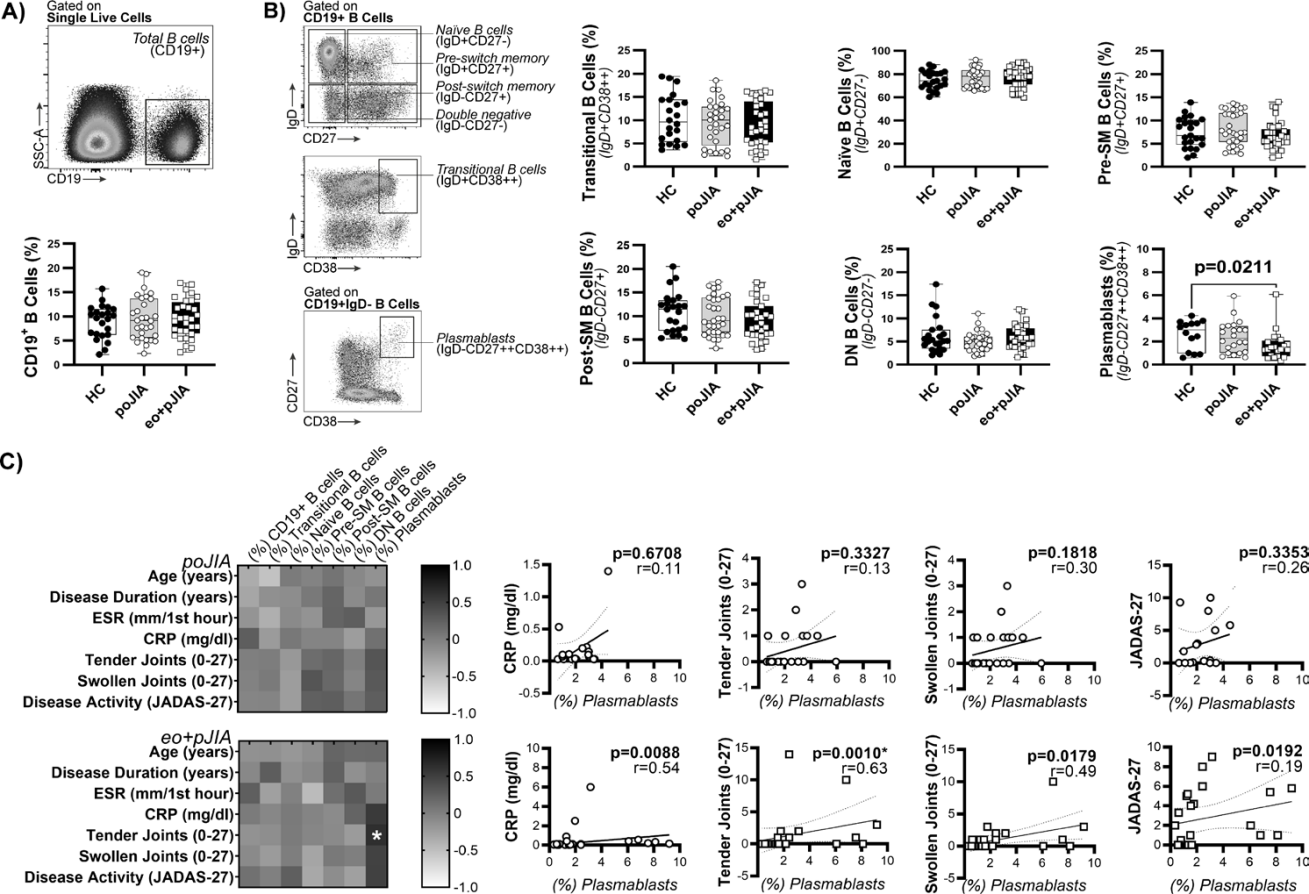
Children with extended oligoarticular and polyarticular JIA, but not persistent oligoarticular JIA, have lower levels of plasmablasts (CD19+IgD-CD27++CD38++) in circulation when compared with healthy controls. The frequency of total CD19+ B cells (A) and B cell subpopulations (B) was determined by flow cytometry in peripheral blood of children with extended oligoarticular and polyarticular JIA when compared with persistent oligoarticular JIA patients and healthy controls. Flow cytometry gating strategy for B cell subpopulations (defined in CD19+ B cells) based on IgD/CD27 and IgD/CD38 classification systems is shown in representative dot plots. Data are represented in box plots. Each dot represents an individual patient. Horizontal lines represent median values, quartiles and extremes (minimum and maximum). Differences were considered statistically significant for p<0.05. Non-parametric Mann-Whitney U test was used for comparisons between 2 independent groups. Kruskal-Wallis test with post hoc Dunn’s multiple comparisons was used to compare more than two groups. A correlation analysis between the frequency of total CD19+ B cells and B cell subpopulations and the clinical data (C) was performed using Spearman correlation test with Bonferroni correction to counteract multiple comparisons and differences were considered statistically significant for p<0.007 (*). Data are represented as heatmap distribution. CRP, C-reactive protein; eo+pJIA, extended oligoarticular and polyarticular JIA; ESR, erythrocyte sedimentation rate; HC, healthy controls; JADAS-27, juvenile arthritis disease activity score 27-joint reduced count; poJIA- persistent oligoarticular JIA.

The frequency of total CD19+B cells in peripheral blood was similar between both groups of children with JIA (persistent oligo JIA and extended oligo+poly JIA) when compared with healthy controls (figure 1A). Children with extended oligo+poly JIA, but not persistent oligo JIA, had significantly lower levels of plasmablasts (CD19+IgD-CD27++CD38++) when compared with controls (figure 1B). No significant differences were observed in the frequencies of transitional B cells (CD19+IgD+CD38++), naïve B cells (CD19+IgD+CD27-), pre-switch memory (pre-SM, CD19+IgD+CD27+), post-switch memory (post-SM, CD19+IgD-CD27+) and double negative (DN, CD19+IgD-CD27-) B cells between both groups of paediatric JIA patients (persistent oligo JIA and extended oligo+poly JIA) in comparison to controls (figure 1B). These observations were also found when analysing absolute B cell numbers (data not shown). Furthermore, no statistically significant correlations were found between the frequencies of total CD19+B cells, transitional B cells, naïve B cells, pre-SM, post-SM and DN B cells and the clinical parameters evaluated (age, disease duration, swollen and tender joint counts, disease activity score (JADAS-27), erythrocyte sedimentation rate (ESR) and C-reactive protein (CRP)) in all patients’ groups (figure 1C). However, we found that, in children with extended oligo+poly JIA, a significant correlation was observed between the frequency of plasmablasts and the number of tender joint counts (figure 1C). No significant differences were found in the frequencies and absolute numbers of total CD19+B cells and B cell subpopulations in adult JIA patients when compared with healthy controls (online supplemental figure 1A). Nonetheless, we found that all children with JIA (extended oligo JIA, poly JIA and persistent oligo JIA) had significantly higher frequencies of transitional and naïve B cells, but lower levels of pre-SM and post-SM B cells when compared with adult JIA patients (data not shown).

10.1136/rmdopen-2022-002901.supp1 Supplementary data

### Children with JIA have alterations in B cell phenotype irrespective of disease category

**Figure 2:**
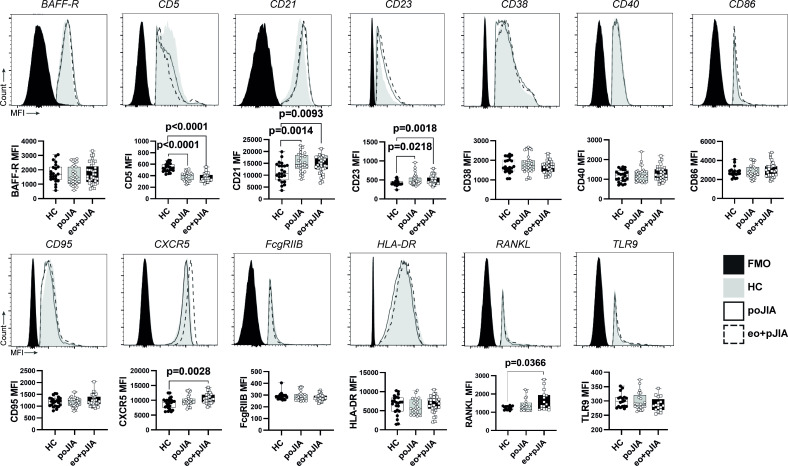
Changes in CD19+ B cells phenotype are found in children with JIA when compared with healthy controls. Phenotypic analysis of total CD19+ B cells in peripheral blood from paediatric JIA patients. The expression levels of B cell activating factor receptor (BAFF-R), activation receptors (CD86, HLA-DR), chemokine receptor (CXCR5), B-cell differentiation (CD5), complement receptor type 2 (CD21), the low-affinity Fc-epsilon-receptor II (CD23), inhibitory receptor (Fc-gamma-RIIB), calcium signalling (CD38), costimulatory receptor (CD40), Fas-receptor (CD95) and toll-like receptor 9 (TLR9) were analysed (median fluorescence intensity, MFI) in total CD19+ B cells to characterise B cell phenotype in children with JIA when compared with healthy controls. Data are represented in box plots. Each dot represents an individual patient. Horizontal lines represent median values, quartiles and extremes (minimum and maximum). Differences were considered statistically significant for p<0.05. Non-parametric Mann-Whitney U test was used for comparisons between two independent groups. Kruskal-Wallis test with post hoc Dunn’s multiple comparison was used to compare more than two groups. eo+pJIA, extended oligoarticular and polyarticular JIA; FMO, fluorescence minus one; HC, healthy controls; JIA, juvenile idiopathic arthritis; poJIA, persistent oligoarticular JIA.

To further investigate B cell abnormalities in JIA, the expression levels (median fluorescence intensity, MFI) of several cellular markers were evaluated on circulating CD19+B cells in this study to characterise B cell phenotype in circulation. We found that children with JIA have an altered B cell phenotype irrespective of disease category (figure 2A). We observed that total CD19+B cells from both groups of children with JIA (persistent oligo JIA and extended oligo+poly JIA) had significantly decreased expression levels of CD5, but increased MFI values of CD21 and CD23 when compared with controls (figure 2). Furthermore, we found that B cells from extended oligo+poly JIA patients, but not persistent oligo JIA, had significantly increased levels of CXCR5 and RANKL in comparison to controls (figure 2). No significant differences were observed in B cell expression levels of BAFF-R, CD38, CD40, CD86, CD95, FcgRIIB, HLA-DR and TLR9 in all paediatric JIA patients when compared with controls (figure 2). In addition, no significant differences were found in the phenotype of total CD19+B cells from adult JIA patients when compared with healthy controls (data not shown).

### An imbalance in circulating Tfh subpopulations, but not in Tfr cells, is observed in children with extended oligoarticular and polyarticular JIA

**Figure 3:**
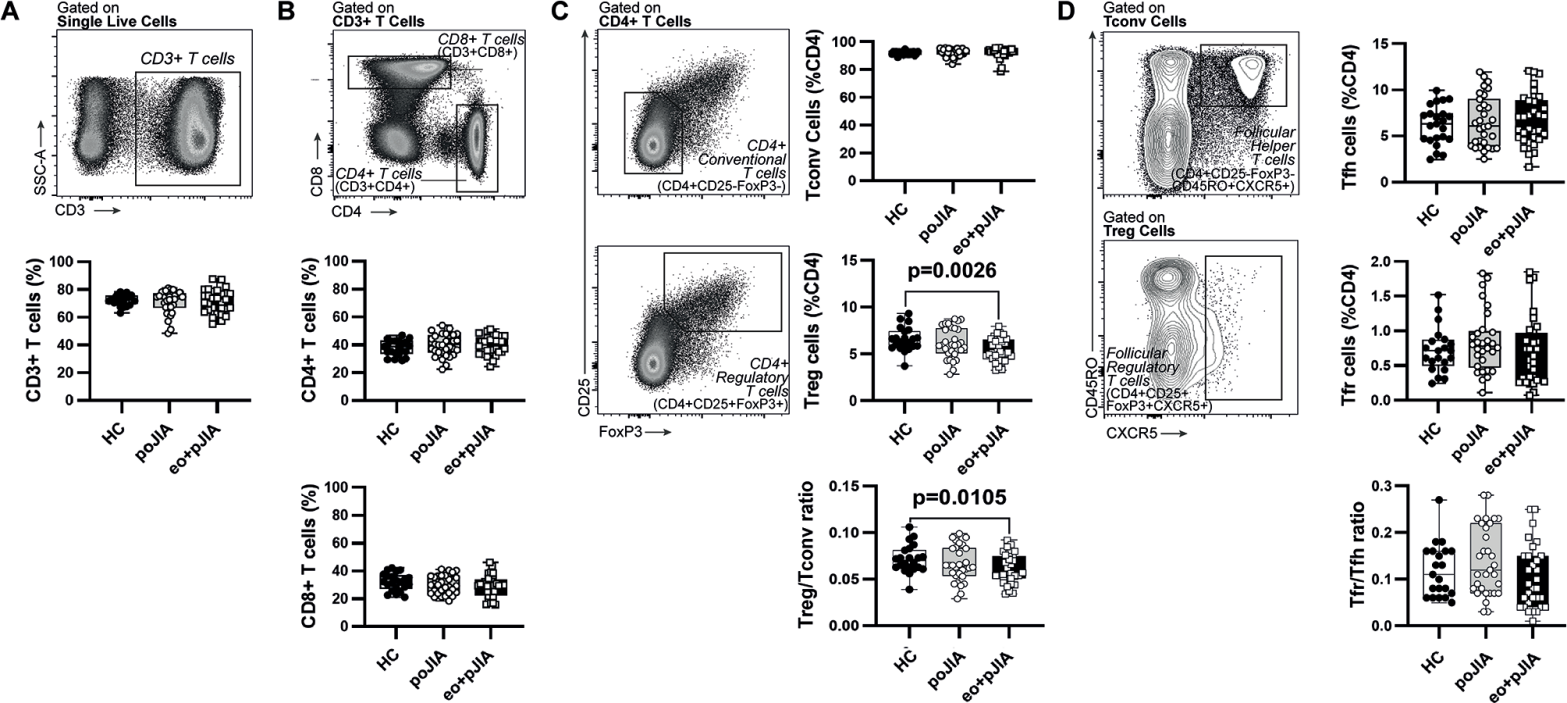
Children with extended oligoarticular and polyarticular JIA, but not persistent oligoarticular JIA have lower levels of regulatory T cells (CD4+CD25+FoxP3+) in circulation when compared with healthy controls. The frequencies of total CD3+ T cells (A), CD4+ and CD8+ T cells (B), CD4+ T conventional (Tconv, CD4+CD25-FoxP3−), regulatory T cells (Tregs, CD4+CD25+FoxP3+) and Tregs/Tconv ratio (C), T follicular helper (Tfh, CD4+CD25-FoxP3-CD45RO+CXCR5+), T follicular regulatory (Tfr, CD4+CD25+FoxP3+CXCR5+) cells and Tfr/Tfh ratio (D), were evaluated by flow cytometry in peripheral blood of children with extended oligoarticular JIA and polyarticular JIA when compared with persistent oligoarticular JIA patients and healthy controls. Data are represented in box plots. Each dot represents an individual patient. Horizontal lines represent median values, quartiles and extremes (minimum and maximum). Differences were considered statistically significant for p<0.05. Non-parametric Mann-Whitney U test was used for comparisons between two independent groups. Kruskal-Wallis test with post hoc Dunn’s multiple comparisons was used to compare more than two groups. eo+pJIA, extended oligoarticular and polyarticular JIA; HC, healthy controls; JIA, juvenile idiopathic arthritis; poJIA, persistent oligoarticular JIA.

**Figure 4:**
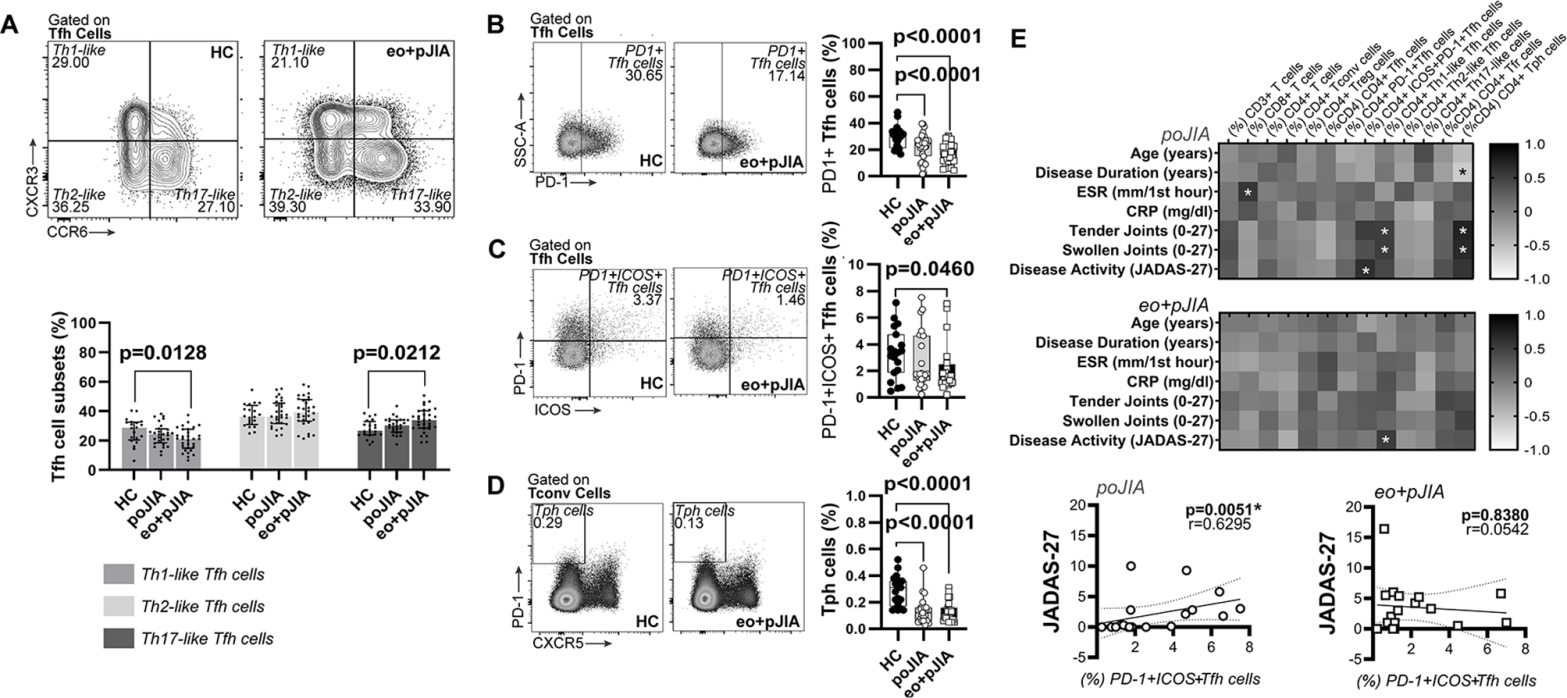
Children with extended oligoarticular and polyarticular JIA, but not persistent oligoarticular JIA have an imbalance in T follicular cell subsets in circulation when compared with healthy controls. The frequencies CD4+ Tfh cell subsets (Tfh1, CXCR3+CCR6−; Tfh2, CXCR3-CCR6−; Tfh17, CXCR3-CCR6+) (A), PD1+ Tfh cells (B), PD1+ICOS+ Tfh cells (C) and Tph (CXCR5-PD-1++) cells (D) were evaluated by flow cytometry in peripheral blood of children with extended oligoarticular JIA and polyarticular JIA when compared with persistent oligoarticular JIA patients and healthy controls. Data are represented in box plots. Each dot represents an individual patient. Horizontal lines represent median values, quartiles and extremes (minimum and maximum). Differences were considered statistically significant for p<0.05. Non-parametric Mann-Whitney U test was used for comparisons between two independent groups. Kruskal-Wallis test with post hoc Dunn’s multiple comparisons was used to compare more than two groups. A correlation analysis between the frequency of total T and T cell subpopulations and clinical data (E) was performed using Spearman correlation test with Bonferroni correction to counteract multiple comparisons and differences were considered statistically significant for p<0.007 (*). Data are represented as heatmap distribution. CRP, C-reactive protein; eo+pJIA, extended oligoarticular and polyarticular JIA; ESR, erythrocyte sedimentation rate; HC, healthy controls; JADAS-27, juvenile arthritis disease activity score 27-joint reduced count; JIA, juvenile idiopathic arthritis; poJIA, persistent oligoarticular JIA.

Paediatric JIA patients had similar frequencies of total CD3+ T cells (figure 3A), CD4+ and CD8+ T cells (figure 3B) when compared with healthy controls (figure 3B). Also, no significant differences were found in the frequency of circulating CD4+ T conventional cells (Tconv, CD4+CD25-FoxP3−) in children with JIA in comparison to controls (figure 3C). However, significantly reduced levels of regulatory T cells (Tregs, CD4+CD25+FoxP3+) were found in peripheral blood from children with extended oligo+poly JIA, but not in persistent oligo JIA patients, when compared with controls (figure 3C). In fact, a significantly reduced Tregs/Tconv ratio was observed in children with extended oligo+poly JIA, but not in persistent oligo JIA patients, when compared with controls (figure 3C). We have also found that children with JIA had similar frequencies of Tfh (CD4+CD25-FoxP3−CD45RO+CXCR5+) and Tfr (CD4+CD25+FoxP3+CXCR5+) cells when compared with healthy controls and no significant differences were detected in Tfr/ Tfh cells ratio (figure 3D). Nevertheless, regarding Tfh cell subpopulations, when using CXCR3/CCR6 expression classification system, we found that children with extended oligo+poly JIA, but not persistent oligo JIA patients, had significantly lower frequencies of Th1-like Tfh (Tfh1, CXCR3+CCR6−) cells and higher frequencies of Th17-like Tfh (Tfh17, CXCR3-CCR6+) cells when compared with controls (figure 4A). Furthermore, we found that PD1+Tfh cells were significantly reduced in JIA patients, irrespective of JIA category, when compared with controls (figure 4B). Moreover, we have also observed that extended oligo JIA and poly JIA patients, but not persistent oligo JIA patients, had significantly lower levels of PD1+ICOS+Tfh cells when compared with controls (figure 4C). Tph cells (CD4+CD25-FoxP3-CXCR5-PD1++) were also evaluated in this study. We found that patients with JIA had lower levels of Tph cells when compared with controls, irrespective of disease category (figure 4D). Overall, these observations were also found when analysing absolute T cell numbers (data not shown). In addition, no statistically significant correlations were found between the frequencies of total CD3+, CD4+, CD4+Tconv, Tregs, Tfh and Tfr cells and the clinical parameters evaluated (age, disease duration, ESR, CRP, swollen and tender joint counts and JADAS-27) in all patients’ groups (figure 4E). A significant correlation was detected between the frequencies of CD8+T cells and ESR in persistent oligo JIA, but not in extended oligo+poly JIA patients (figure 4E). Moreover, we have also observed that the frequencies of PD1+ICOS+Tfh cells and Th1-like Tfh cells were significantly correlated with JADAS-27 in persistent oligo JIA and extended oligo+poly JIA patients, respectively (figure 4E) No major significant differences were found in the frequencies and absolute numbers of total CD3+T cells and their subpopulations in adult JIA patients when compared with healthy controls (online supplemental figure 1B). No significant differences were found regarding PD1+Tfh and Tph cells in adult JIA when compared with heathy controls (data not shown).

### An altered phenotype of Tfh cells is detected in children with JIA irrespective of disease category

**Figure 5:**
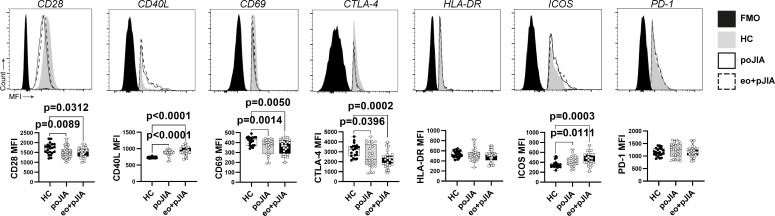
Changes in Tfh cells phenotype are found in children with JIA when compared with healthy controls. A phenotypic analysis of circulating Tfh (CD4+CD25-FoxP3−CD45RO+CXCR5+) cells was performed by flow cytometry in peripheral blood from paediatric JIA patients. The expression levels of T-cell costimulatory receptor CD28, CD40 ligand (CD40-L), programmed cell death protein 1 (PD-1), inducible T-cell costimulator (ICOS), cytotoxic T lymphocyte antigen-4 (CTLA-4) and activation markers (CD69, HLA-DR) were analysed (median fluorescence intensity, MFI) in Tfh (CD4+CD25-FoxP3-CD45RO+CXCR5+) cells to characterise Tfh cell phenotype in children with JIA when compared with healthy controls. Data are represented in box plots. Each dot represents an individual patient. Horizontal lines represent median values, quartiles and extremes (minimum and maximum). Differences were considered statistically significant for p<0.05. Non-parametric Mann-Whitney U test was used for comparisons between two independent groups. Kruskal-Wallis test with post hoc Dunn’s multiple comparisons was used to compare more than two groups. eo+pJIA, extended oligoarticular and polyarticular JIA; FMO, fluorescence minus one; HC, healthy controls; JIA, juvenile idiopathic arthritis; poJIA, persistent oligoarticular JIA.

To characterise Tfh (CD4+CD25-FoxP3-CD45RO+CXCR5+) cells phenotype in circulation the expression levels (MFI) of several cellular markers were analysed in this study. We found a reduced activated phenotype of Tfh cells in children with JIA, irrespective of disease category (figure 5). We observed that Tfh cells from both groups of children with JIA (persistent oligo JIA and extended oligo+poly JIA) had significantly lower expression levels of CD28, CD69 and CTLA-4, but increased expression levels of CD40L and ICOS when compared with controls (figure 5). No significant differences were observed in the expression levels of HLA-DR and PD-1 in both groups of paediatric JIA patients analysed when compared with controls (figure 5). No major significant differences were found in the phenotype of Tfh cells in adult JIA patients when compared with healthy controls (data not shown).

### Children with extended oligoarticular and polyarticular JIA, but not persistent oligoarticular JIA patients, have a cytokine pattern in circulation sustaining B cell activation

**Figure 6:**
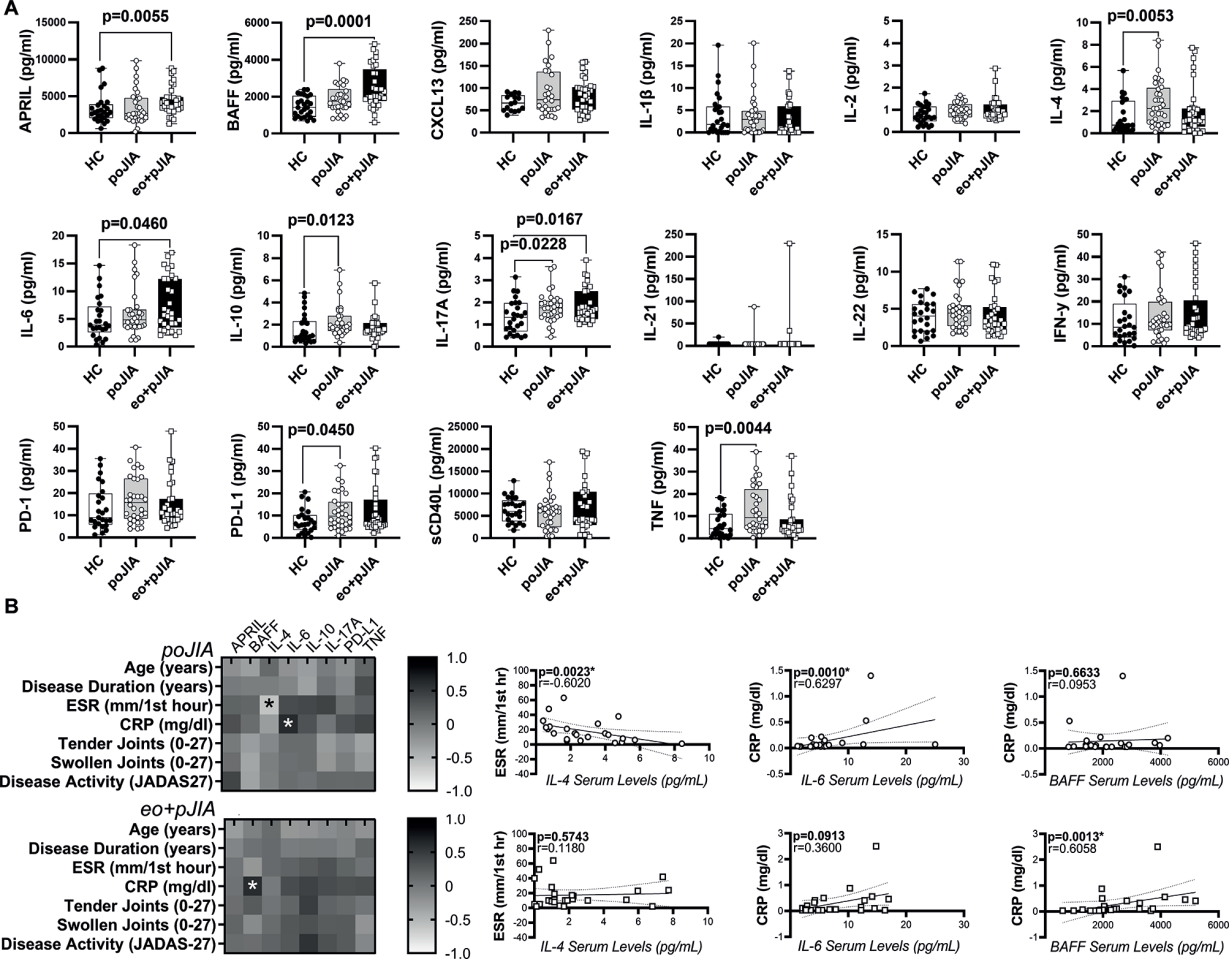
Children with extended oligoarticular and polyarticular JIA, but not persistent oligoarticular JIA patients, have a cytokine pattern in circulation sustaining B cell activation. Serum levels of A proliferation-inducing ligand (APRIL), B cell activating factor (BAFF), interleukin (IL)-1β, IL-2, IL-4, IL-6, IL-10, IL-17A, IL-21, IL-22, interferon gamma (IFN-γ), programmed cell death-protein 1 (PD-1), programmed death-ligand 1 (PD-L1), soluble CD40 ligand (sCD40L), chemokine (C-X-C motif) ligand 13 (CXCL13) and tumour necrosis factor (TNF) were measured by multiplex bead-based immunoassay and/ or ELISA in children with extended oligoarticular and polyarticular JIA when compared with persistent oligoarticular JIA patients and healthy controls (A) Data are represented in box plots. Each dot represents an individual patient. Horizontal lines represent median values, quartiles and extremes (minimum and maximum). Differences were considered statistically significant for p<0.05. Non-parametric Mann-Whitney U test was used for comparisons between two independent groups. Kruskal-Wallis test with post hoc Dunn’s multiple comparisons was used to compare more than two groups. A correlation analysis between cytokine serum levels and clinical data (B) was performed using Spearman correlation test with Bonferroni correction to counteract multiple comparisons and differences were considered statistically significant for p<0.007 (*). Data are represented as heatmap distribution. CRP, C-reactive protein; eo+pJIA, extended oligoarticular and polyarticular JIA; ESR, erythrocyte sedimentation rate; HC, healthy controls; JADAS-27, juvenile arthritis disease activity score 27-joint reduced count; JIA, juvenile idiopathic arthritis; poJIA, persistent oligoarticular JIA.

To investigate the underlying mechanisms of B and T cell alterations observed in paediatric JIA patients, we have evaluated the cytokine environment present in circulation in all groups included. We found that children with extended oligo+poly JIA, but not persistent oligo JIA patients, had significantly increased serum levels of APRIL, BAFF and IL-6 when compared with healthy controls (figure 6A). Contrarily, paediatric persistent oligo JIA patients, but not extended oligo+poly JIA, had significantly higher serum levels of IL-4, IL-10, sPD-L1 and TNF when compared with controls (figure 6A). In addition, IL-17A serum levels were significantly elevated in all paediatric JIA groups when compared with controls (figure 6A). Furthermore, no significant differences were observed in the remaining cytokines quantified (IL-1β, IL-2, IL-21, IL-22, IFN-γ, CXCL13, PD-1 and sCD40L) between all paediatric JIA groups analysed in comparison with controls (figure 6A). Moreover, no significant differences were detected between children with extended oligo JIA, poly JIA and persistent oligo JIA. No significant correlations were found between the cytokine serum levels and clinical data (age, disease duration, ESR, CRP, tender and swollen joint counts and JADAS-27) in children with extended oligo+poly JIA, except a significant correlation detected between BAFF serum levels and CRP values (figure 6B). In children with persistent oligo JIA, significant correlations were found between IL-4 serum levels and ESR, and IL-6 serum levels and CRP values (figure 6B). Of note, no significant correlations were observed between cytokine serum levels and the frequencies of B and T cell subpopulations in circulation, nor with B and T cell markers expression levels (data not shown). Additionally, no major significant differences were detected in the cytokine panel analysed in serum samples from adult JIA patients when compared with healthy controls (online supplemental figure 1C).

## Discussion

In this study, we found that children with extended oligo JIA and poly JIA have alterations in B cells, Tregs and T follicular cell subsets in peripheral blood and a cytokine profile sustaining B cell activation. Previous studies have demonstrated that alterations in B cell subpopulations, particularly memory B cell subsets, occur in JIA patients, not only in peripheral blood, but also locally in the joints.^22 24–27 42 43^ In particular, Corcione *et al*^22^ have documented enriched levels of plasmablasts in synovial fluid when compared with paired peripheral blood samples of JIA patients, especially in extended oligo JIA comparing to persistent oligo JIA. Herein, lower levels of plasmablasts (CD19+IgD-CD27++CD38++) found in peripheral blood of extended oligo JIA and poly JIA patients indicate an abnormal distribution of B cell subsets, which might be related with B cell retention or trafficking towards the inflamed tissues and/ or with infiltration of the synovial membrane. In addition, we found that children with JIA have alterations in B cell phenotype irrespective of disease category. Overall, B cell phenotype analysis of JIA patients suggests altered B cell activating triggering mechanisms (CD5, CD21 and CD23), higher trafficking susceptibility (CXCR5) and triggering of bone erosion (RANKL).^44 45^ Regarding T cells, several studies support a role of T cells in JIA pathogenesis. Changes in T cell homoeostasis, differentiation and function have been described in circulation and locally in the joints.^46–53^ Particularly, disturbances in Tregs were observed in JIA.^54^ Accordingly, we found that children with extended oligo JIA and poly JIA have lower levels of Tregs (CD4+CD25+FoxP3+) in circulation when compared with controls, suggesting that a breakdown of Treg-mediated peripheral tolerance might occur in JIA pathogenesis. However, the knowledge about Tfh and Tfr cells in JIA pathophysiology is still scarce.^48 55^ Tfh and Tfr cells are primarily found in secondary lymphoid organs, but a small percentage recirculates in the blood. Circulating Tfh cells differentiate into distinct phenotypically subsets, identified by the expression of CXCR3 and CCR6 (Th1-like, Th2-like and Th17-like Tfh cells). Nonetheless, only Th2-like and Th17-like Tfh cells are able to activate B cells and promote production of immunoglobulins.^56^ In this study, we found an imbalance in circulating Tfh cell subsets, but not in Tfr cells, in children with extended oligo JIA and poly JIA in comparison to healthy controls. Although no significant differences were observed regarding the frequency of Tfh (CD4+CD25-FoxP3-CD45RO+CXCR5+) and Tfr (CD4+CD25+FoxP3+CXCR5+) cells, we found that Th1-like Tfh (CXCR3+CCR6−) cells were significantly decreased in extended oligo JIA and poly JIA patients, contrarily to Th17-like (CXCR3-CCR6+) Tfh cells that were significantly increased when compared with controls. These observations suggest a role of Th17-like Tfh cells in the pathogenesis of extended oligo JIA and poly JIA. In fact, previous studies have demonstrated that JIA patients have a predominant Th17 cells’ phenotype not only in circulation, but also in joints, which has been associated with JIA pathophysiology and reinforces a Th17 polarisation environment.^57^ Indeed, a Th17/Treg cell imbalance has been suggested as a contributor to JIA pathogenesis.^46 58 59^ Furthermore, lower levels of PD1+Tfh cells, PD1+ICOS+Tfh cells and Tph cells were found in JIA patients when compared with controls. Although the frequencies observed of Tfh and Tph cells are not consistent among distinct immune-mediated diseases,^60^ some studies described an increased frequency of these T cell subpopulations in peripheral blood and synovial fluid from JIA patients.^48–50^ In particular, it was observed that Tph cells are enriched in the joints of oligo JIA patients and in the synovial fluid of ANA+ JIA patients, where they express many B cell helper-associated markers and have the capacity to induce B cell differentiation and antibody production.^50^ Unlike Tfh cells, Tph cells provide help to B cells within inflamed tissues, such as joints, and therefore, might constitute and important player in JIA pathogenesis.^61^ In our study, the lower levels of activated Tfh cells and Tph cells might suggest an active recruitment of these cells to inflammatory sites such as the synovial membrane, where an interaction with infiltrating B cell subsets can occur and thus contribute to disease physiopathology.^49 50^ Nevertheless, a treatment effect cannot be excluded. In addition, a reduced activated phenotype (CD28 and CD69) of Tfh (CD4+CD25-FoxP3-CD45RO+CXCR5+) cells was observed in JIA patients irrespective of disease category. These findings might be a result of the treatment effect on Tfh cell activation. Nevertheless, lower expression levels of CTLA-4 and higher expression levels of ICOS and CD40L on Tfh were also found in JIA patients, which might support a potentially relevant interplay between Tfh and B cells in JIA pathogenesis. In fact, ICOS expression is crucial for GC formation, and CD40L can induce B cell proliferation and activation.^62^ Furthermore, we have also found that children with extended oligo JIA and poly JIA, but not persistent oligo JIA, have a cytokine pattern in peripheral blood that supports B cell activation. Previous studies have described changes in serum and synovial fluid levels of several proinflammatory cytokines in JIA patients.^63–65^ Also, different genetic polymorphisms of cytokine genes have been associated to JIA.^66 67^ Herein, we found elevated serum levels of APRIL, BAFF and IL-6 in extended oligo JIA and poly JIA patients when compared with healthy controls, similarly to what has been previously described.^12 65 68^ These results suggest a potential role of these cytokines in JIA pathogenesis, considering their relevance in B cell activation, differentiation and function and, therefore, reinforce B cell contribution in JIA pathophysiology.^69^ Of interest, prior results from our group demonstrated that a cytokine pattern favouring B-cell activation and survival is present in RA patients since the first weeks of disease development.^12^ Furthermore, we have also shown for the first time that alterations in B cell subpopulations are detected in peripheral blood in very early RA patients.^11 14^ Thus, as hypothesised, the results obtained in the present study support similarities between extended oligo JIA/poly JIA and RA.^5^ In addition, considering that IL-6 also promotes the differentiation of Tfh cells, our results might indicate an altered homoeostasis and/or activation profile of Tfh cell subpopulations. Moreover, regarding IL-17A, our findings are in line with previous studies by our group and others.^10 64 70^ IL-17A is a potent proinflammatory cytokine, produced mainly by Th17 cells, that has been found to be highly expressed in the inflamed synovium and strongly contributes to the production of several other proinflammatory cytokines (such as IL-6 and RANKL), therefore amplifying the inflammatory cascade and contributing to tissue destruction.^71 72^ The elevated IL-17A serum levels found in this study support a role of this cytokine, Th17 and/or Th17-like Tfh cells in JIA development. Interestingly, similar observations were published by our group in early RA.^10–14^ Overall, this study suggests that B cells, Tregs and follicular T cell subsets, particularly Th17-like Tfh cells, might be associated to the pathogenesis of extended oligo JIA and poly JIA, in line with previous observations obtained in adults with early RA,^10–14^ thus reinforcing clinical evidence showing that these JIA categories tend to evolve into a RA like phenotype.^5^ Nevertheless, it remains unclear whether the observed B and Tfh cell alterations are a cause or a consequence of the humoral immune dysregulation underlying JIA. Future studies exploring the role of these cells in JIA are necessary to more accurately determine their relevance in JIA pathogenesis. In adult JIA patients, no major significant differences were observed in the frequency and phenotype of B and T cell subpopulations, or in the cytokine profile in peripheral blood when compared with healthy controls. These results might be related with long-term treatment effects. Of note, it has been previously described by our group that, overall, adult JIA patients have lower functional impairment and better quality of life than patients with adult-onset rheumatic diseases,^6^ which might be related to a lower immunological and inflammatory ongoing burden in adult JIA patients. Nevertheless, significant differences were observed between children and adult JIA patients. We found that children with JIA had higher frequencies of transitional and naïve B cells, but lower levels of pre-SM and post-SM B cells when compared with adult JIA. This is probably related to an immature immune system present in children when compared with adults.^73^ This study has some limitations that need to be considered in data interpretation. Treatment effect cannot be excluded since JIA patients enrolled in this study were treated with synthetic and/or biologic DMARDs which have been suggested to affect B and T cell numbers, differentiation stage, proliferation capability and apoptosis in JIA.^55 74^ Thus, future studies focused in untreated extended oligo JIA and poly JIA patients should be considered. Furthermore, disease activity might also be a confounder, since the paediatric groups of JIA patients included in our study had mostly low to moderate disease activity or were in remission according to JADAS-27 score. Therefore, future studies with a higher number of patients included in each disease activity subgroup would allow a more robust statistical analysis and a more accurate evaluation of disease activity effect. In addition, due to comprehensive ethical limitations in blood collection of paediatric patients, it was not possible to have a sufficient number of isolated cells to perform in vitro functional assays to better analyse the function of B and follicular T cell subsets in JIA patients, which should be pursued in future research studies. To sum up, our results suggest that children with extended oligo JIA and poly JIA, but not persistent oligo JIA patients, have alterations in B and T follicular cell subsets in peripheral blood and a cytokine profile sustaining B cell activation. In particular, the lower levels of plasmablasts, Tregs and increased frequencies of Th17-like Tfh cells detected in extended oligo JIA and poly JIA patients might suggest a potential contribution of these cells in the pathogenesis of these JIA categories.

10.1136/rmdopen-2022-002901.supp2 Supplementary data

10.1136/rmdopen-2022-002901.supp3 Supplementary data

10.1136/rmdopen-2022-002901.supp4 Supplementary data

## Data Availability

All data relevant to the study are included in the article or uploaded as online supplemental information. The data underlying this article will be shared on reasonable request to the corresponding author RAM.
