# Supplementary material for: Children with extended oligoarticular and polyarticular juvenile idiopathic arthritis have alterations in B and T follicular cell subsets in peripheral blood and a cytokine profile sustaining B cell activation
Source: RMD Open. 2023 Aug 31;9(3):e002901. doi: 10.1136/rmdopen-2022-002901 (PMC10476142; doi:10.1136/rmdopen-2022-002901)
Supplement: Supplementary data [file rmdopen-2022-002901supp001.pdf]

## MATERIALS AND METHODS

### Clinical Assessment and Patient Recruitment

A total of 105 JIA patients, including pediatric (<18 years old, n=68) and adult (≥18 years old, n=37) JIA patients were enrolled in this study. Blood samples were collected from pediatric patients with extended oligoarticular JIA and polyarticular JIA followed up at the Pediatric Rheumatology Unit, Hospital de Santa Maria, Centro Hospitalar Universitário Lisboa Norte (CHULN), Centro Académico de Medicina de Lisboa (CAML); Hospital Garcia de Orta and Centro Hospitalar de Lisboa Ocidental (CHLO), Portugal. Pediatric patients with persistent oligoarticular JIA were also included as a disease control. Patients with other forms of JIA were excluded from the study. Adult oligoarticular and polyarticular JIA patients followed up at the Rheumatology Department, Hospital de Santa Maria, CHULN, CAML, Portugal were also recruited for comparison. All patients recruited were treated with non-steroidal anti-inflammatory drugs (NSAIDs), synthetic and/ or biologic disease modifying anti-rheumatic drugs (DMARDs). Patients' characterization, including age, gender, disease duration, disease activity measured by juvenile arthritis disease activity score 27-joint reduced count (JADAS-27) or disease activity score of 28 joints (DAS28), functional status (childhood health assessment questionnaire, CHAQ), treatment and clinical evaluation of antinuclear antibodies (ANA), rheumatoid factor (RF) and anti-citrullinated protein antibodies (ACPA) were collected from the Rheumatic Diseases Portuguese Register (Reuma.pt) (www.reuma.pt)[1]. Inactive disease was defined in pediatric patients with a cut-off JADAS-27 ≤1 and in adults with a cut-off DAS28 ≤2.6. In addition, two groups of age-matched healthy individuals (children and adults) were included as controls. Healthy children and adolescents were recruited at the Department of Pediatric Surgery and at the Division of Adolescent Medicine, Hospital Santa Maria, CHULN, CAML, Portugal. Children and adolescents with acute or chronic inflammatory states were excluded. Trained health care professionals collected the samples to minimize child pain, distress and/ or fear. The Biobank facility at Instituto de Medicina Molecular João Lobo Antunes (IMM) (Biobanco-IMM), Faculdade de Medicina, Universidade de Lisboa and Centro de Investigação Clínica (CIC) at Hospital Santa Maria, CHULN, CAML, Portugal supported the collection and/ or storage of human samples necessary to the development of this study. Written informed consent was obtained from all patients and healthy individuals and/ or from the patients' parents/ legal guardians, prior to any protocol-specific procedure. This study protocol has been conducted in compliance with the Declaration of Helsinki as amended in Fortaleza, Brazil (2013) and it was approved by the CAML Ethics Committee.

**PBMC Isolation**

Fresh peripheral blood mononuclear cells (PBMC) were isolated from heparinized whole blood samples following density gradient centrifugation with Lymphocyte Separation Medium (LSM) (Corning, USA). Cells were washed twice in 1X phosphate buffered saline (PBS) and cellular viability was estimated with 0.4% Trypan Blue (Sigma, USA) exclusion method.

**Immunophenotyping of B and T cell subpopulations**

The frequency and phenotype of peripheral blood B and T cell subpopulations were evaluated by flow cytometry on freshly isolated PBMC samples in all groups included. The gating strategy used to identify all B and T cell subpopulations analyzed in this study is described in *Supplementary Figure 2*. Total B cell (CD19+) subpopulations were classified according to IgD/CD27 and IgD/CD38 classification systems as transitional (IgD+CD38++); naïve (IgD+CD27-); pre-switch memory (pre-SM, IgD+CD27+); post-switch memory B cells (post-SM, IgD-CD27+); double negative B cells (DN, IgD-CD27-) and plasmablasts (IgD-CD27++CD38++). The expression of B cell activating factor receptor (BAFF-R), activation receptors (CD86, HLA-DR), chemokine receptors (CXCR5), complement receptor type 2 (CD21), the low-affinity Fc-epsilon-receptor II (CD23), inhibitory receptor (Fc-gamma-RIIB, FcgRIIB), costimulatory receptor (CD40), Fas-receptor (CD95), toll-like receptor (TLR)-9, receptor activator of nuclear factor kappa-B ligand (RANKL) and CD5 was analyzed in all B cell subsets. Total T cell (CD3+) subpopulations were classified as cytotoxic T cells (CD8+); helper T cells (CD4+); regulatory T cells (CD4+CD25+FoxP3+); follicular T helper cells (CD4+CD25-FoxP3-CXCR5+CD45RO+); follicular T helper type 1-like cells (CXCR5+CXCR3+CCR6-); follicular T helper type 2-like cells (CXCR5+CXCR3-CCR6-); follicular T helper type 17-like cells (CXCR5+CXCR3-CCR6+); follicular T regulatory cells (CD4+CD25+FoxP3+CXCR5+) and peripheral helper T cells (CD4+CD25-FoxP3-CXCR5-PD-1++). The expression of T-cell costimulatory receptor CD28, CD40 ligand (CD40-L), programmed cell death protein 1 (PD-1), inducible T-cell costimulator (ICOS), cytotoxic T lymphocyte antigen-4 (CTLA-4) and activation markers (CD69, HLA-DR) was analyzed in all T cell subsets. For cell surface staining, PBMC were incubated with monoclonal antibodies directly conjugated to fluorochromes and fixed with IC Fixation Buffer (eBioscience, USA). For CTLA-4, FoxP3 and TLR-9 intracellular staining, FoxP3 Fix/Perm Kit (eBioscience, USA) was used according to manufacturer’s instructions. All antibodies were purchased from BioLegend (USA), BD Biosciences (USA) and ThermoFisher Scientific (USA). A minimum of 100 000 cells gated in lymphocytes/ sample were acquired with LSR Fortessa™ Cell Analyzer (BD Biosciences, USA) using FACSDiva software (BD Biosciences, USA). Viable cells were defined by Fixable Viability Dye (ThermoFisher Scientific, USA) staining exclusion. Eight peak calibration Rainbow Beads (BD

Biosciences, USA) were used to ensure stable fluorescence measurements throughout the study. Data were analyzed with FlowJo (TreeStar, Stanford University, California, USA). Absolute cell counts were calculated from differential leukocyte counts determined for all patients and healthy controls.

**Cytokine and chemokine quantification**

Serum levels of A proliferation-inducing ligand (APRIL), B cell activating factor (BAFF), interleukin (IL)-1β, IL-2, IL-4, IL-6, IL-10, IL-17A, IL-22, interferon gamma (IFN-γ), programmed cell death-protein 1 (PD-1), programmed death-ligand 1 (PD-L1), soluble CD40 ligand (sCD40L) and tumor necrosis factor (TNF) were measured by multiplex bead-based immunoassay (LEGENDplex™, BioLegend, USA) according to the manufacturer's instructions. Samples were processed using LSR Fortessa™ Cell Analyzer (BD Biosciences, USA) and data files were analyzed using LEGENDplex™ Data Analysis Software. Additionally, serum levels of chemokine (C-X-C motif) ligand 13 (CXCL13) (R&D Systems, United Kingdom) and IL-21 (BioLegend, USA) were quantified by enzyme-linked immunosorbent assay (ELISA) according to the manufacturer's instructions. Samples were analyzed using plate reader Infinite M200 (Tecan, Switzerland).

**Statistical Analysis**

Statistical analyses were performed using GraphPad Prism (GraphPad, San Diego, USA). Categorical variables were expressed as frequencies and differences were tested using Chi-Square test. For continuous variables in populations that did not follow a Gaussian distribution, non-parametric tests were used. Mann-Whitney U test was used to compare two independent groups. Kruskal-Wallis test with post-hoc Dunn's multiple comparisons was used to compare more than two groups. Correlation studies of quantitative variables were performed using Spearman's correlation coefficient test, followed by Bonferroni correction to counteract multiple comparisons. Outliers of the study were excluded by calculating the mean ± 2 standard deviation (SD). All differences were considered statistically significant for P-values < 0.05 or 0.007 (with Bonferroni correction).

**REFERENCES:**

1 Canhao H, Faustino A, Martins F, *et al.* Reuma.pt - The Rheumatic Diseases Portuguese Register. Acta Reumatol Port. 2011; 36:45–56.
