# Supplementary figures and images for: Children with extended oligoarticular and polyarticular juvenile idiopathic arthritis have alterations in B and T follicular cell subsets in peripheral blood and a cytokine profile sustaining B cell activation
Source: RMD Open. 2023 Aug 31;9(3):e002901. doi: 10.1136/rmdopen-2022-002901 (PMC10476142; doi:10.1136/rmdopen-2022-002901)

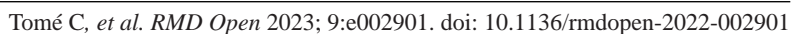

Supplement: Supplementary data [file rmdopen-2022-002901supp002.pdf]

A) B cells gating strategy

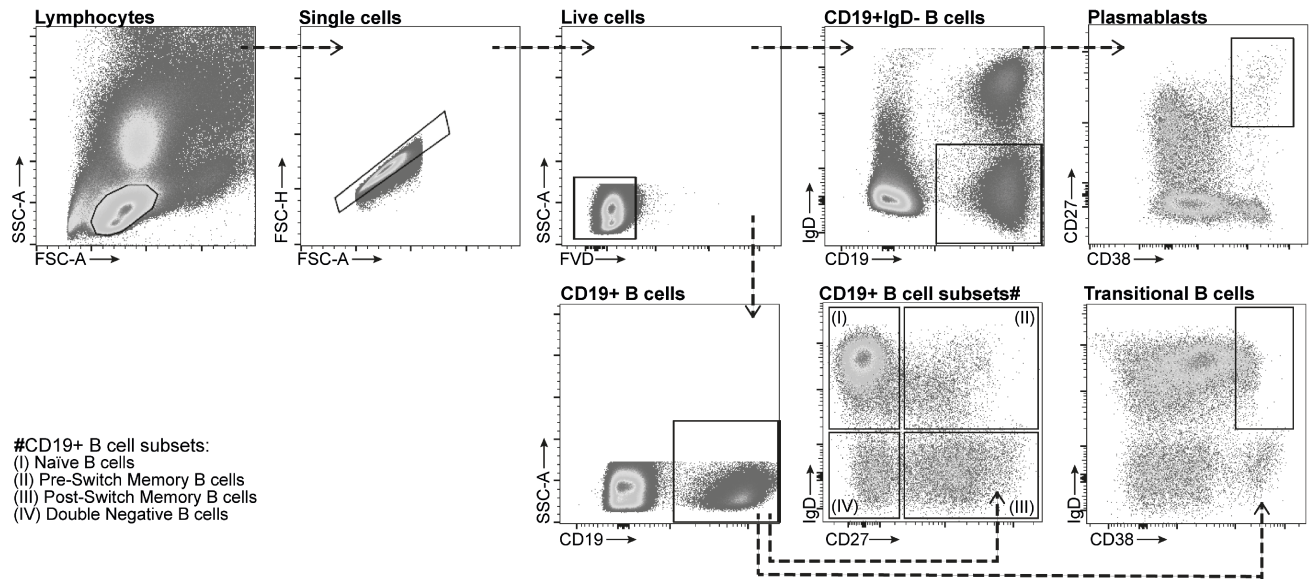

B) T cells gating strategy

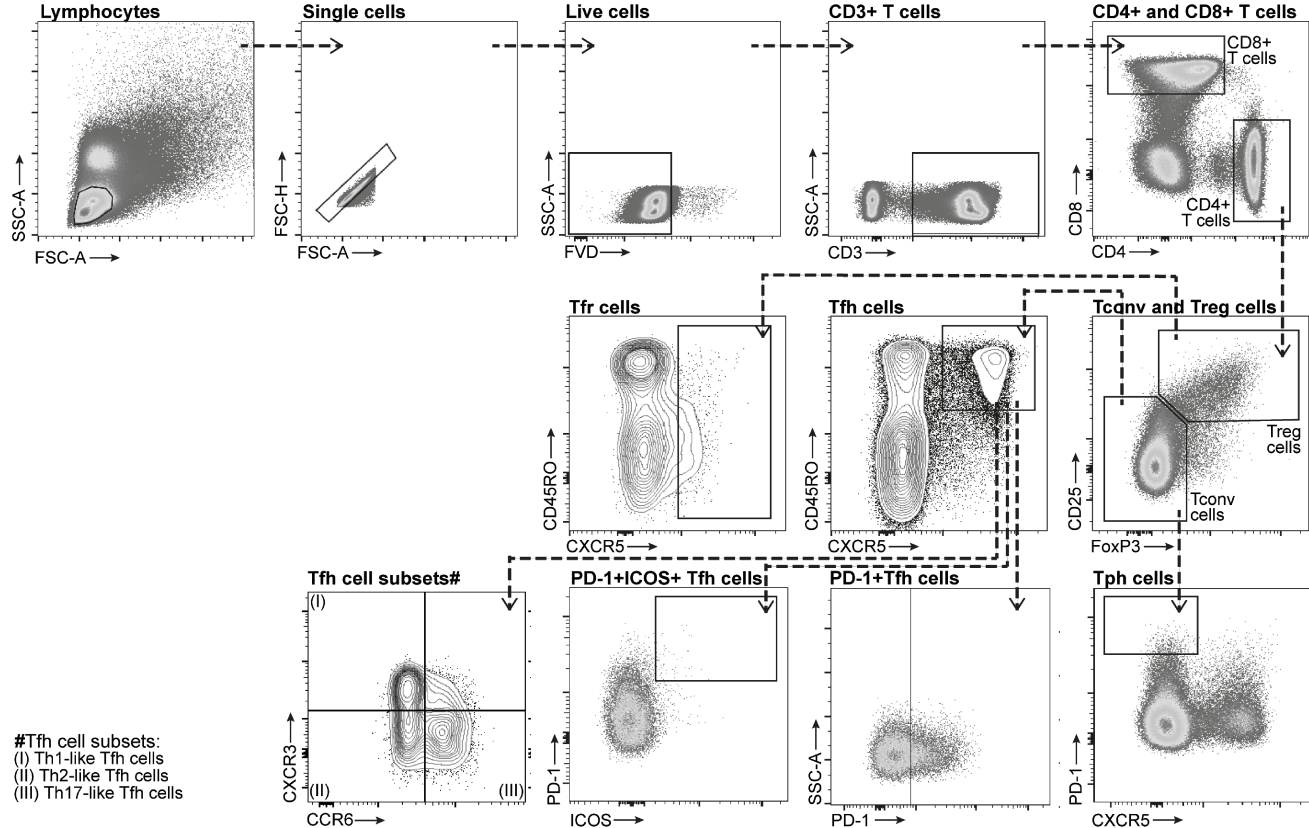

Supplement: Supplementary data [file rmdopen-2022-002901supp003.pdf]
