# Supplementary material for: Children with extended oligoarticular and polyarticular juvenile idiopathic arthritis have alterations in B and T follicular cell subsets in peripheral blood and a cytokine profile sustaining B cell activation
Source: RMD Open. 2023 Aug 31;9(3):e002901. doi: 10.1136/rmdopen-2022-002901 (PMC10476142; doi:10.1136/rmdopen-2022-002901)
Supplement: Supplementary data [file rmdopen-2022-002901supp004.pdf]

SUPPLEMENTARY FIGURE LEGENDS

Suppl. Fig. 1 – **Adult JIA patients have no major alterations in the distribution of B and T cell subpopulations and in the cytokine profile in circulation when compared to healthy controls**. The frequency of total CD19+ B cells, B cell subpopulations (A), total CD3+ T cells and T cell subpopulations (B) was determined by flow cytometry. Serum levels of A proliferation-inducing ligand (APRIL), B cell activating factor (BAFF), interleukin (IL)-1β, IL-2, IL-4, IL-6, IL-10, IL-17A, IL-21, IL-22, interferon gamma (IFN-γ), programmed cell death-protein 1 (PD-1), programmed death-ligand 1 (PD-L1), soluble CD40 ligand (sCD40L), chemokine (C-X-C motif) ligand 13 (CXCL13), and tumor necrosis factor (TNF) were measured by multiplex bead-based immunoassay and/ or ELISA (C). The experiments were performed in peripheral blood of adults with extended oligoarticular and polyarticular JIA when compared to persistent oligoarticular adult JIA patients and healthy controls. Flow cytometry gating strategy for B and T cell subpopulations is shown in representative dot plots. Data are represented in box plots. Each dot represents an individual patient. Horizontal lines represent median values, quartiles, and extremes (minimum and maximum). Differences were considered statistically significant for $p<0.05$. Non-parametric Mann-Whitney U test was used for comparisons between 2 independent groups. Kruskal-Wallis test with post-hoc Dunn's multiple comparisons was used to compare more than two groups. CRP - C-reactive protein; eo+pJIA - extended oligoarticular and polyarticular JIA; ESR - erythrocyte sedimentation rate; HC - healthy controls; JADAS27 - juvenile arthritis disease activity score 27-joint reduced count; poJIA - persistent oligoarticular JIA.

Suppl. Fig. 2 – **Gating strategy used to define B cell subsets (A) and T cell subpopulations (B) in peripheral blood.** Total B cells were identified as CD19+ lymphocytes and B cell subpopulations were defined using IgD/CD27 and IgD/CD38 classification systems as transitional (IgD+CD38++); naïve (IgD+CD27-); pre-switch memory (pre-SM, IgD+CD27+); post-switch memory B cells (post-SM, IgD-CD27+); double negative B cells (DN, IgD-CD27-) and plasmablasts (IgD-CD27++CD38++). Total T cells were identified as CD3+ lymphocytes and were classified into CD4+ T cells or CD8+ T cells. CD4+ T cells were further classified as cytotoxic T cells (CD8+); helper T cells (CD4+); regulatory T cells (CD4+CD25+FoxP3+); follicular T helper cells (CD4+CD25-FoxP3-CXCR5+CD45RO+); follicular T helper type 1-like cells (CXCR3+CCR6-); follicular T helper type 2-like cells (CXCR3-CCR6-); follicular T helper type 17-like cells (CXCR3-CCR6+); activated Tfh subsets (PD-1+ and PD-1+ICOS+); follicular T regulatory cells (CD4+CD25+FoxP3+CXCR5+) and peripheral helper T cells (CD4+CD25-FoxP3-CXCR5-PD-1++).

Tomé C, *et al. RMD Open* 2023; 9:e002901. doi: 10.1136/rmdopen-2022-002901
